# Virulence of the *Pseudomonas fluorescens* clinical strain MFN1032 towards *Dictyostelium discoideum* and macrophages in relation with type III secretion system

**DOI:** 10.1186/1471-2180-12-223

**Published:** 2012-09-29

**Authors:** Daniel Sperandio, Victorien Decoin, Xavier Latour, Lily Mijouin, Mélanie Hillion, Marc G J Feuilloley, Nicole Orange, Annabelle Merieau

**Affiliations:** 1 LMSM, Laboratoire de Microbiologie Signaux et Micro-Environnement, EA 4312, Université de Rouen, 55 rue Saint Germain, Evreux, 27000, France

**Keywords:** *Pseudomonas fluorescens* clinical strains, Type III secretion system, *Dictyostelium discoideum*, Macrophage necrosis, Cell-associated hemolytic activity

## Abstract

**Background:**

*Pseudomonas fluorescens* biovar I MFN1032 is a clinical isolate able to grow at 37°C. This strain displays secretion-mediated hemolytic activity involving phospholipase C and cyclolipopeptides, and a cell-associated hemolytic activity distinct from the secreted hemolytic activity. Cell-associated hemolysis is independent of biosurfactant production and remains in a *gacA* mutant. Disruption of the hrpU-like operon (the basal part of type III secretion system from rhizospheric strains) suppresses this activity. We hypothesized that this phenotype could reflect evolution of an ancestral mechanism involved in the survival of this species in its natural niche. In this study, we evaluated the hrpU-like operon’s contribution to other virulence mechanisms using a panel of *Pseudomonas* strains from various sources.

**Results:**

We found that MFN1032 inhibited the growth of the amoebae *Dictyostelium discoideum* and that this inhibition involved the hrpU-like operon and was absent in a *gacA* mutant. MFN1032 was capable of causing macrophage lysis, if the hrpU-like operon was intact, and this cytotoxicity remained in a *gacA* mutant. Cell-associated hemolytic activity and macrophage necrosis were found in other *P. fluorescens* clinical isolates, but not in biocontrol *P. fluorescens* strains harbouring hrpU-like operon. The growth of *Dictyostelium discoideum* was inhibited to a different extent by *P. fluorescens* strains without correlation between this inhibition and hrpU-like operon sequences.

**Conclusions:**

In *P. fluorescens* MFN1032, the basal part of type III secretion system plays a role in *D. discoideum* growth inhibition and macrophage necrosis. The inhibition of *D. discoideum* growth is dependent on the GacS/GacA system, while cell-associated hemolytic activity and macrophage lysis are not. Virulence against eukaryotic cells based on the hrpU-like operon may be more than just a stochastic evolution of a conserved system dedicated to survival in competition with natural predators such as amoebae. It may also mean that there are some important modifications of other type III secretion system components, which remain unknown. Cell-associated hemolysis might be a good indicator of the virulence of *Pseudomonas fluorescens* strain.

## Background

*Pseudomonas fluorescens* is a highly heterogeneous species of γ Proteobacteria [1,2]. Saprophytic members of this species are found in large numbers in all of the major natural environments and also form associations with plants [3-5]. Surprisingly, *P. fluorescens* includes some strains suspected to be opportunistic human pathogens [6,7]. Recently, and despite its psychrotrophy (optimal growth temperature range between 25–30°C) [8], several studies highlighted the infectious potential of some *Pseudomonas fluorescens* clinical strains [9-11]. MFN1032 is a clinical strain, identified as belonging to biovar I of *P. fluorescens* species, which was isolated from a patient with a lung infection and is able to grow at 37°C [11]. We previously described that MFN1032 cells induce necrosis and apoptosis in rat glial cells at this temperature. This strain adheres to intestinal epithelial cells where it induces cytotoxic effects and proinflammatory reactions [12]. MFN1032 displays secretion-mediated hemolytic activity involving phospholipase C and cyclolipopeptides [13]. This activity is positively regulated by the two-component system GacS/GacA and is subject to phase variation [9,14]. MFN1032 shows a cell-associated hemolytic activity distinct from the secreted hemolytic activity. The cell-associated hemolytic activity (cHA) is expressed at 37°C and is detected *in vitro* in mid log growth phase in the presence of erythrocytes. This cHA is independent of phospholipase C and cyclolipopeptide production and increases in a *gacA* mutant. GacS/GacA seems to be a negative regulator of this activity. Finally, MFN1032 harbours type III secretion system (T3SS) genes [15]. In *Pseudomonas aeruginosa* CHA strain, cell-associated hemolytic activity is correlated with secretion of PcrV, PopB and PopD by T3SS. This pore forming activity precedes macrophage oncosis [16]. In addition, numerous studies have reported the implication of T3SS in the infectivity of *P. aeruginosa* in *Dictyostelium discoideum*. *D. discoideum* is a soil amoeba that feeds on bacteria by phagocytosis [17,18]. It was used as a model eukaryotic cell, which mimics mammalian macrophage in how it interacts with microbes. *P. aeruginosa* can kill *D. discoideum* by delivering effector proteins to target cells [19,20].

T3SS genes are absent from the *P. fluorescens* Pf0-1 and Pf5 genomes published in databases [21,22] but are present in numerous plant-associated and biocontrol *P. fluorescens* strains [23-26]. Strain KD protects the cucumber from the oomycete *Pythium ultimum,* and its T3SS, acquired horizontally from phytopathogenic bacteria, decreases pectinase polygalacturonase activity (a key pathogenicity factor) from *P. ultimum*[26]. This strain does not induce a Hypersensitivity Response (HR) on tobacco leaves. In C7R12 and SBW25, two other biocontrol strains with T3SS genes, the target of T3SS has not been fully elucidated [25,27]. In *P. fluorescens* Q8r1-96, T3SS is different from its counterparts in SBW25 and similar to *P. syringae* T3SS. This strain expresses T3SS effectors capable of suppressing HR [23].

MFN1032 possesses some contrasting features of saprophytic or pathogenic *Pseudomonas* in regards to T3SS. MFN1032 has T3SS-like genes, *hrcRST*, with a high level of homology to the *hrcRST* genes of the hrpU operon in *Pseudomonas syringae* DC3000. Disruption of this hrpU-like operon in MFN1032 abolishes cell-associated hemolytic activity [15], as described for mutations in the T3SS apparatus in *P. aeruginosa*. Our hypothesis was that the first target of MFN1032 T3SS would probably be eukaryotic cells of the rhizosphere, such as plants or amoebae.

To test this hypothesis, we investigated the interactions of MFN1032 and other *Pseudomonas* strains with red blood cells, plants, amoebae and macrophages. In contrast with environmental *Pseudomonas*, all of the clinical strains of *P. fluorescens* tested were cytotoxic for erythrocytes through contact. MFN1032 was unable to induce HR on plants and was cytotoxic for amoebae and macrophages. Disruption of the hrpU-like operon in MFN1032 abolished these cytotoxicities that were independent of cyclolipopeptide production. GacS/GacA system seems to be a positive regulator for *D. discoideum* growth inhibition but not for cell-associated hemolysis or macrophage lysis, suggesting that these processes are not identical.

## Results

### *P. fluorescens* MFN1032 and other clinical strains have cell-associated hemolytic activity but do not induce HR on tobacco leaves

We investigated the distribution of cell-associated hemolytic activity on a panel of *Pseudomonas* strains. Cell-associated hemolytic activity (cHA) was measured by the technique used by Dacheux [16], adapted as described in methods. We tested cHA at 37°C for MFN1032, MFY162, MFY70 and MFY63 (clinical isolates of *P. fluorescens)*, MF37 (*P. fluorescens* strain isolated from raw milk), C7R12 and SBW25 (rhizospheric *P. fluorescens* strains) and DC3000 (*P. syringae* plant pathogen) after growth at 28°C (for strain origin see Table 1). 

**Table 1:** **Bacterial strains used inthis study, origins, growthtemperatures and references**

**Species**	**Strains**	**Optimal growth temperature (°C)**	**Origins**	**References**
*Pseudomonas fluorescens*	SBW25	28°C	Field grown-sugar beet	[25]
	C7R12		Flax rhizosphere	[27]
	MF37		Milk tank	[39]
	MFY63		Clinical (urine)	[6]
	MFY70		Clinical (abscess)	[6]
	MFY162		Clinical (sputum)	[6]
	MFN1032		Clinical (sputum)	[11]
	MFN1030		MFN1032 hrpU-like operon mutant	[15]
	MFN1030- pBBR1MCS-5		MFN1030 carrying pBBR1MCS-5	This study
	MFN1030-pBBR-*rsc*STU		MFN1030 carrying *rsc*STU genes of SBW25 cloned into pBBR1MCS-5	This study
	MFN1031		MFN1030 revertant	[15]
	V1		MFN1032 spontaneous *gacA* mutant	[9]
	V1*gac*A		V1 carrying the *gacA* gene (plasmid pMP5565)	[9]
	V3		MFN1032 Variant group 2 (Cyclolipopeptides -)	[9,14]
*Pseudomonas syringae*	DC3000		Tomato	[40]
*Pseudomonas aeruginosa*	CHA	37°C	Clinical	[41]
	PA14		Clinical	[42]
*Klebsiella aerogenes*	KA		Environmental	[43]

**Figure 1:**
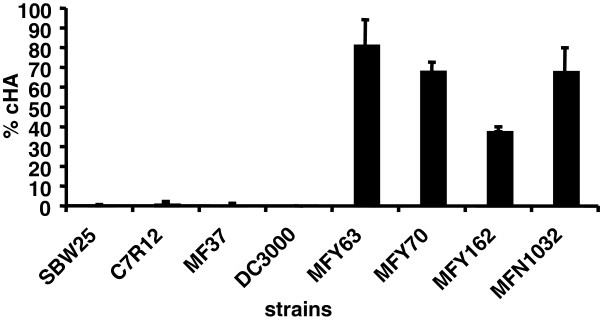
** Cell-associated hemolytic activity (cHA).** Cell-associated hemolytic activity (cHA) was measured as described in the materials and methods. Results are mean values from at least three independent experiments. Standard deviation is shown. RBCs were incubated 1h at 37°C with MFN1032, MFY63, MFY70, MFY162, SBW25, C7R12, MF37 or DC3000 cultivated at 28°C (MOI of 1).

Only clinical strains had cHA (Figure 1). MFY63 showed the highest level of cHA (80% lysis); MFY70 and MFN1032 displayed significant cHA (70% lysis) and MFY162 a median cHA (40% lysis). In the case of the environmental strains tested, C7R12, SBW25, MF37 and DC3000 were not hemolytic.

**Figure 2:**
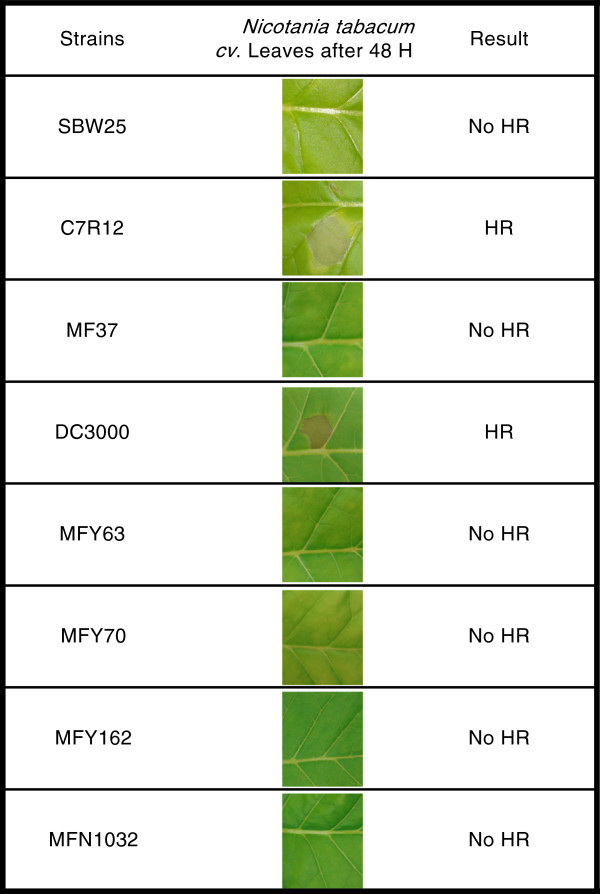
** Plant hypersensitive response (HR)assay. ***P. fluorescens* strains, MFN1032, MFY63, MFY70, MFY162, SBW25, C7R12, MF37 and *P. syringae* DC3000, were infiltrated into *Nicotiana tabacum cv.* leaves. The leaves were evaluated for production of HR and were photographed after 48 h. This experiment was repeated 2 times with similar results.

The same panel of strains was tested on tobacco leaves to determine if these strains were able to induce HR. As illustrated in Figure 2, HR was only detected for C7R12 and DC3000. All clinical strains i.e., MFY63, MFY70, MFY162 and MFN1032 and two environmental strains, SBW25 and MF37, were unable to induce HR.

### *P. fluorescens* MFN1032 is virulent on *Dictyostelium discoideum* (*D. discoideum)*

**Figure 3:**
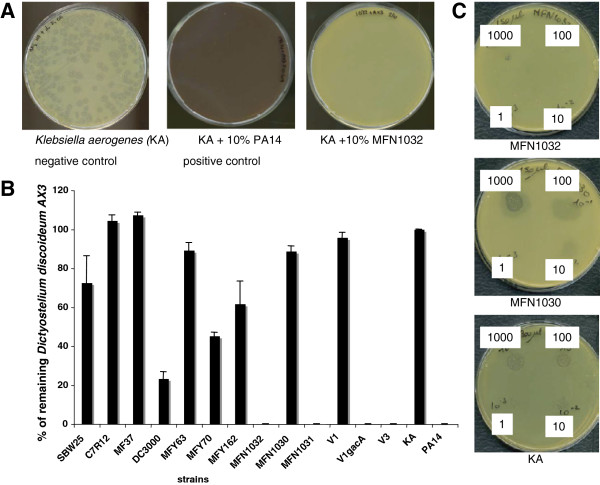
** Virulence towards *Dictyostelium discoideum.*** Approximately 100 *D. discoideum* cells were cultivated in SM-plates with the indicated proportion of *Klebsiella aerogenes* and *Pseudomonas* strains (10%). Plates were maintained at 22°C for 5 days. **A:***Pseudomonas aeruginosa* PA14 (positive control), *Klebsiella aerogenes* (KA, negative control) and *P. fluorescens* MFN1032 virulence towards *D. discoideum* after 5 days. **B:** Virulence of different *Pseudomonas* strains at 10% against *D. discoideum*. These results were obtained by the ratio of the number of lysis plaques obtained with the negative control *Klebsiella aerogenes* (100% of amoebae remained). Standard deviation is shown. Data are mean values from three independent experiments. **C**: *D. discoideum* growth on layer of MFN1032, MFN1030 or KA as described in the materials and methods. 1000, 100, 10 and 1 indicated number of *D. discoideum* per μL.

As described in Figure 3A, *Klebsiella aerogenes* (KA) (negative control for virulence), *Pseudomonas aeruginosa* PA14 (positive control for virulence), and MFN1032 were tested on *D. discoideum*. On a layer of KA, about one hundred lysis plaques were observed, corresponding to the zone where actively feeding and replicating *D. discoideum* have phagocytosed the bacteria. On a layer of PA14 or MFN1032 at 10%, no lysis plaque was detected. MFN1032 does indeed display a virulent phenotype on *D. discoideum*, either by evading *D. discoideum* killing*,* or by actively killing amoebae. Then, our panel of strains was tested on *D. discoideum* (Figure 3B). Two strains, C7R12 and MF37 had a complete absence of *D. discoideum* growth inhibition (100% of *D. discoideum* remained). MFY63 and SBW25 were highly permissive for *D. discoideum* growth (90% and 75% of amoebae remained, respectively). MFY70 and MFY162 permitted the replication of about half of the *D. discoideum* (40% and 60% respectively). DC3000 had a slightly virulent phenotype on *D. discoideum* (20% of *D. discoideum* remained). In our panel, to small to be representative, *D. discoideum* growth inhibition above 50% was only observed for clinical or phytopathogenic strains of *Pseudomonas*.

*P. fluorescens* MFN1032 virulence towards *D. discoideum* is dependent on the hrpU-like operon and the GacS/GacA two-component system and is independent of cyclolipopeptides (CLPs).

We used a mutant strain, MFN1030, the hrpU-like operon mutant of MFN1032, to determine whether T3SS apparatus proteins are required for the MFN1032 phenotype with respect to *D. discoideum*. MFN1030 was permissive for *D. discoideum* growth (90% of *D. discoideum* remained). The revertant of MFN1030, MFN1031, inhibited D*. discoideum* growth.

We investigated the possible involvement of the GacS/GacA two-component system in the regulation of this phenotype using a *gacA* spontaneous mutant of MFN1032, V1. V1 is defective for cyclolipopeptide (CLP) production and secreted hemolysis, but still exhibits cHA. V1 was plated on *D. discoideum* and allowed these amoebae to grow, as described in Figure 3B (100% of *D. discoideum* remained). Introduction of a *gacA* gene in V1, to give the V1*gacA* strain, restored wild-type phenotype*.*

CLP biosurfactant production is positively regulated by the GacS/GacA system in numerous *P.fluorescens* strains [9,28]. Biosurfactants produced by *P. aeruginosa* have been reported to cause the lysis of *D. discoideum*[20]. To investigate the role of CLP, we took advantage of strain V3, a MFN1032 variant (described as a “group 2 variant”), which have a defect in CLP production but which have a wild type GacS/GacA [9,14]. V3 does not show other measurable modifications from secreted factors. V3 inhibited fully *D. discoideum* growth (0% of amoebae remained).

*D. discoideum* growth inhibition could be due to MFN1032-induced death of *Klebsiella aerogenes*, which is the feeding source of the amoeba. To exclude this possibility, we counted *Klebsiella aerogenes* colony forming unit (CFU) after 5 days at 22°C in SM medium, either with or without the presence of MFN1032, MFN1030 or V1. In all conditions, the *Klebsiella aerogenes* counts were identical (approximately 10^8^ CFU.mL^-1^).

Moreover, as described in Figure 3 C, MFN1030 as sole feeding source permitted *D. discoideum* growth in 2 days at 22°C, while MFN1032 did not. Similar results were obtained with V1 (Data not shown).

### *P. fluorescens* MFN1032 is cytotoxic on macrophages via intracellular mechanisms

**Figure 4:**
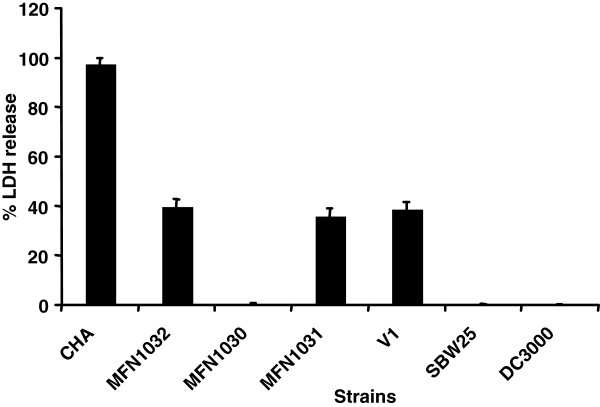
** Cytotoxic activity on macrophage J774A. 1.** J774A.1 macrophages grown in 24-well plates for 20 h were infected with strains grown to an OD_580nm_ of 1.0-1.5 (MOI of 5). The cytotoxicity was followed over a 4 h period by measuring LDH release using a cytotoxicity detection kit (Promega). Values are expressed as a mean concentration of LDH in the culture after 4 h of incubation. Data are mean values from three independent experiments.

In order to correlate *D. discoideum* growth inhibition (which mimic macrophage phagocytosis) and cytotoxicity towards macrophages, we infected cell line J774A.1 macrophages with MFN1032 (not permissive), DC3000 (slightly not permissive) and SBW25 (highly permissive) as described in Material and Methods. The strain of *P. aeruginosa* CHA is a clinical isolate from a patient suffering from cystic fibrosis and has been used as a positive control for macrophage lysis, monitored by LDH release [29]. This strain has a highly inducible T3SS, responsible for virulence behaviour [30]. This strain provoked full lysis of macrophages in our conditions (Figure 4). MFN1032 displayed an LDH release of 40% whereas SBW25 and DC3000 were unable to lyse macrophages. These results showed that, in DC3000, slight virulence towards *D. discoideum* is not correlated with macrophage necrosis. 

In order to determine the possible involvement of T3SS in macrophage lysis by MFN1032, we used MFN1030 (hrpU-like operon mutant) to infect J774A.1 macrophages. MFN1030 was impaired in macrophage lysis whereas MFN1031 (MFN1030 revertant) had a wild type phenotype with a 40% LDH release. The *gacA* mutant of MFN1032, V1, had the same range of macrophage lysis as MFN1032 (Figure 4).

**Figure 5:**
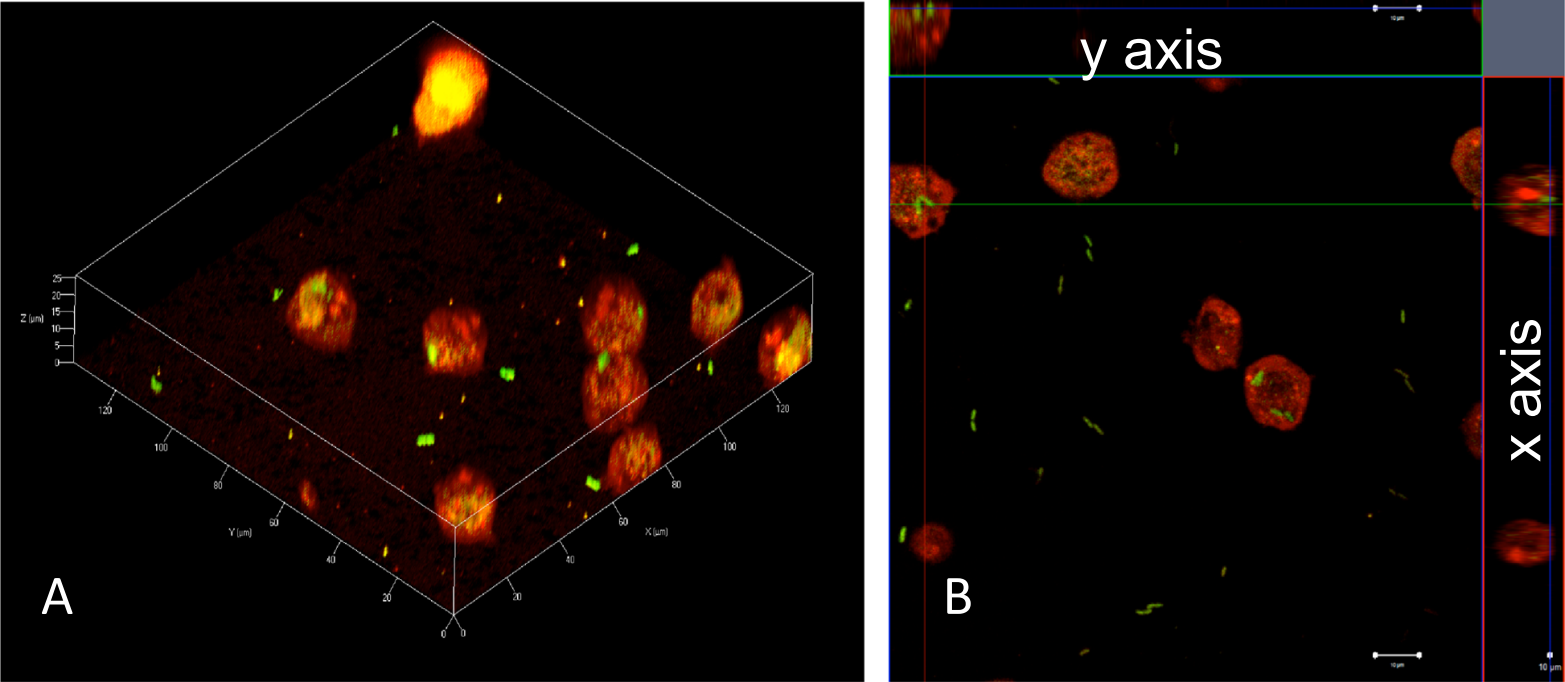
** In vivo microscopy ofmacrophages infected by MFN1032.** Confocal laser-scanning photography of *Pseudomonas fluorescens* MFN1032 with J774A.1 macrophages. J774A.1 macrophages grown in 24-well plates for 20h were infected with strains grown to an OD_580nm_ of 1.0-1.5 (MOI of 10). Cytotoxicity was followed over a 10 min period by *in vivo* microscopy. The dead macrophages were red (by EtBr entry) and MFN1032 expressing GFP were green. **A**: Representative photography of a 3D modelisation of 17 z stack images of 1μm. **B**: Representative orthographic representation of 1μm thick layer. The cell at the crossing of the red and green lines in the z stack has been submitted to a stack in the x and y axis.

Confocal analysis of macrophages infected by MFN1032 was conducted to study this necrosis. Following ten minutes of infection, numerous macrophages appeared red in medium containing EtBr, confirming a rapid necrosis (Figure 5A). Orthographic representation revealed that every dead macrophage contained MFN1032 expressing green fluorescent protein (Figure 5B). Only few live macrophages, which were not stained but perceptible by their autofluorescence, contained intracellular bacteria (data not shown).

### MFN1030 (hrpU-like operon disrupted mutant) phenotypes can be partially restored by expression of hrpU-like operon genes from SBW25

MFN1030 is a mutant containing an insertion that disrupts the hrpU-like operon. This strategy of mutation can cause polar effects, i.e genetic modifications outside the targeted region. Thus, the phenotypes observed could be related to genes other than the hrpU-like operon. To check this possibility, the *rsc*STU genes of SBW25 (corresponding to the *hrc*STU genes of MFN1032 affected by the hrpU-like operon disruption) were expressed from plasmid pBBR1MCS-5 in MFN1030. We choose to clone the *rsc*STU genes of SBW25 for complementation experiments because SBW25 genome is sequenced (in contrast to the *hrcU* gene of MFN1032) and the *rsc*RST genes present more than 90% of identity with the *hrc*RST genes of MFN1032. The phenotypes of the resulting strain, MFN1030-pBBR-*rsc*STU, are summarised in Table 2 (Results are means of at least three independent experiments). *D. discoideum* growth inhibition and cHA were restored in MFN1030-pBBR-*rsc*STU, with levels similar to those characteristic of wild type MFN1032. Macrophages lysis was partly restored in MFN1030-pBBR-*rsc*STU with a level corresponding to half of that of the wild type. Introduction of parental plasmid pBBR1MCS-5 in MFN1030 (MFN1030-pBBR1MC-5 strain) did not modify MFN1030 phenotypes.

**Table 2:** **Phenotypes of MFN1032, MFN1030,MFN1030-pBBR-*rsc*STU and MFN1030-pBBR1MCS-5**

**Phenotypes**	**Strains**			
	**MFN1032**	**MFN1030**	**MFN1030-pBBR-*rsc*STU**	**MFN1030-pBBR1MCS-5**
Cell-associated hemolytic activity (% cHA at 28°C)	69 ± 10	9 ± 7	69 ± 3	12 ± 4
*D. discoideum* growth inhibition (%)	100	11 ± 3	100	9 ± 2
Macrophages lysis (% LDH release)	40 ± 3	0	24 ± 2	0

## Discussion

### cHA seems dependent on strain origin and not only on T3SS basal part homology

All clinical *P. fluorescens* strains had cHA while environmental strains of *Pseudomonas* did not. Nevertheless, hrpU-like operons of SBW25, MF37 (environmental strains) and MFN1032 are highly homologous (more than 90% identity for the HrcR protein) [15]. This was confirmed by complementation of MFN1030 by the SBW25 genes. Even if hrpU-like operon genes are essential to the cHA of MFN1032, as demonstrated by MFN1030 mutant and complementation results, other factors that depend on the origin of the strain, like the T3SS upper part components or the T3SS effectors, are necessary for red blood cell lysis.

In C7R12 and SBW25 the functionality or mechanism of T3SS are not fully understood. On the contrary, *P. syringae* DC3000 has a functional T3SS with HrpZ as a translocation protein. In our conditions, T3SS of this phytopathogen was not able to induce cHA. This result confirms the inability of HrpZ to cause RBC lysis as described by Lee [31]. Moreover, none of the clinical strains induced HR on tobacco leaves, while C7R12 did. This suggests that the hrpU-like operons have a function in the hemolytic *P. fluorescens* clinical strains different from that in the biocontrol and phytopathogenic strains, which are able to induce T3SS mediated HR. These findings are in concordance with those of Mavrodi *et al.* who demonstrated the presence of stable divergent lineages of T3SS in *Pseudomonas fluorescens* strains [23].

### *P. fluorescens* clinical strains inhibit *D. discoideum* growth

*D. discoideum* growth inhibition is not a common feature in this species and was rarely found in *P. fluorescens* environmental strains, even if our panel is too low to be representative. The majority of environmental *P. aeruginosa* isolates have functional T3SSs with toxins that facilitate killing amoebae, their natural predators. Their T3SSs may have evolved for this purpose and broad conservation of targeted substrates across eukaryotic organisms resulted in a system active against human cells [32]. In *P. fluorescens*, the T3SS distribution is not homogenous. hrpU-like operons were absent from Pf0-1 and Pf5 but were present in numerous other rhizospheric strains [22,24], which leads us to believe that this mechanism of resistance to *D. discoideum* predation are not essential to *P.fluorescens* survival. However, the natural niches of *P. fluorescens* and *P. aeruginosa* are mainly the same, and bacteria are exposed to the same predation by amoebae. It should be noted that this it is, to our knowledge, the first report of *P. fluorescens* strains virulence towards amoebae.

### *D. discoideum* growth inhibition by MFN1032 seems positively controlled by the GacS/GacA system and involves the hrpU-like operon

An interesting result was the loss of MFN1032 virulence towards *D. discoideum* in *gacA* and in hrpU-like operon mutants. Involvement of GacS/GacA in growth inhibition of *D. discoideum* has been reported in a strain of *P. entomophila,* a soil bacterium with cyclolipopeptide production. *P. entomophila gacA* mutant is avirulent but CLPs and T3SS were not involved in virulence [33]. In *P. aeruginosa* full virulence requires T3SS and quorum sensing molecules (under GacS/GacA control) [18,20]. Again, these results underline the similarity of mechanisms with *P. aeruginosa*, despite the phylogenetic distance between the T3SS basal parts of these two species.

### Macrophage necrosis required the hrpU-like operon and is independent of the GacS/GacA system

MFN1032 was able to provoke macrophage lysis in our conditions, but it was only half has effective as the CHA strain, a highly pathogenic *P. aeruginosa* strain*.* Macrophages lysis was not fully restored in the complemented strain, MFN1030-pBBR-*rsc*STU. That could be the consequence of the expression of *rsc*STU genes from a plasmid, under P*lac* promotor control, without their own upstream regulatory sequences. As with the CHA strain, necrosis was rapid (less than 10 minutes) for some macrophages. All dead macrophages contained bacteria. We hypothesize that bacterial internalisation by phagocytosis activity is a signal for an induction of virulence factor secretion. This rapid necrosis required hrpU-like operon and was independent of the GacS/GacA two-component system. These dependencies suggest that this mechanism is different from *D. discoideum* growth inhibition and similar to cHA activity. This was confirmed by the results in DC3000 which was unable to lyse macrophages and partially able to resist *D. discoideum* predation but lacking in cHA. The mechanism of DC3000 virulence towards *D. discoideum* is to our knowledge unknown. Some literature suggests that this activity could be due to the action of biosurfactants produced by this strain [34].

## Conclusions

MFN1032 is able to induce macrophage and red blood cell lysis and to prevent *D. discoideum* predation. In these three processes, hrpU-like operon is required but GacA/GacS positive regulation concerns only the *D. discoideum* model. Our findings establish a link between the T3SS and virulence of MFN1032 against eukaryotic cells. This study also underlines the high heterogeneity of the *Pseudomonas* according to their origin. The hypothesis of virulence acquisition towards human cells by a stochastic evolution of an ancestral mechanism dedicated to natural predator, such as amoebae, cannot explain all our results. We suggest that a major evolution of upper T3SS compounds or T3SS toxins, despite the conservation of the T3SS basal part, could be at the origin of MFN1032 virulence. This work must be extended to a larger representative panel of *Pseudomonas fluorescens* strains to confirm this hypothesis.

## Methods

### Cell associated hemolytic activity assay (cHA)

The cHA assay was done essentially as described by Dacheux [16]. Sheep red blood cells (RBC), obtained from Eurobio (France), were washed three times in PBS (pH 7.2, 0.8% NaCl, 0.02% KCl, 0.17% Na_2_HPO_4_, 0.8% KH_2_PO_4_) and resuspended in RPMI-1640 medium without pH indicator (Sigma) at a density of 5 × 10^8^ RBC mL^-1^ at 4°C. The bacteria were grown in LB to an OD_580nm_ of 0.7 – 1.5, centrifuged and resuspended in RPMI-1640 at 5 × 10^8^ bacteria.mL^-1^. Hemolysis assays were started by mixing 100 μL of RBC and 100 μL of bacteria, which were then centrifuged at 400 g for 10 minutes and incubated at 37°C for 1 h. The release of hemoglobin was measured at 540 nm, after centrifugation, in 100 μL of cell supernatant.

The percentage (%) of total lysis was calculated as follows: $\%=\left[\left(\text{X}-\text{B}\right)/\left(\text{T}-\text{B}\right)\right]\times 100$, where B (baseline), a negative control, corresponds to RBC incubated with 100 μL of RPMI-1640, and T, a positive control, corresponds to total RBC lysis, obtained by incubating cells with 0.1% SDS. X is the OD value of the analysed sample.

### Plant Hypersensivity Response (HR) assay

Plant HR assay was done essentially as described by Guo [35]. Bacterial strains grown on King B plates were resuspended at 1 x 10^8^ cell.mL^-1^ in 5 mM MES (Morpholineethane-sulfonic acid) pH 5.6. Each bacterial strain tested was infiltrated in *Nicotiana tabacum* cv. Xanthi. HR were recorded after 24 to 48 h.

### *Dictyostelium discoideum* growth and plating assays

This test was performed essentially as described by Carilla-Latorre [36]. *Dictyostelium discoideum* AX3 cells were grown axenically in HL5 medium pH 6.5 (Formadium) or in association with *Klebsiella aerogenes* on SM plates pH 6.5 (Formadium).

For the nutrient SM-plating assay, *P. fluorescens* strains, *P. aeuginosa* PA14 (positive control of virulence) and *Klebsiella aerogenes* (KA) (negative control of virulence) were grown overnight in LB. After washing in HL5, the tested bacteria were resuspended with HL5 to an optical density of 1 at 580 nm (1 OD_580nm_) and KA was adjusted to 0.5 OD_580nm_.

300 μL of KA and 15 μl of *Pseudomonas* (ratio 10%) were plated in SM-agar plates with approximately 100 *D. discoideum* cells. The plates were maintained at 22°C for 5 days.

KA count were realized after incubation of 300 μL of KA with or without 15 μL of MFN1032, MFN1030 or V1 (ratio 10%) in SM at 22°C for 5 days. Serial dilutions were plated on Hektoen enteric agar (bioMerieux) at 37°C to select KA.

For some assay, 150 μL of MFN1032, MFN1030, V1 (0.5 OD_580nm_) or 300 μL of KA (1 OD_580nm_) were plated in SM-agar plates and 2 μL of serial dilution of *D. discoideum* culture (respectively 1000,100, 10 or 1 *D. discoideum* per μL) were spotted on the bacterial layer. The plates were maintained at 22°C for 2 days.

### Cell culture and infection conditions

Macrophage cell line J774A.1 was grown in Dulbecco’s modified Eagle Minimal Essential Medium (DMEM) (Lonza) containing 10% foetal calf serum (FCS) supplemented with 2 mM L-glutamine, 100 μg.mL^-1^ penicillin, 100 μg.mL^-1^ streptomycin and 2 mM pyruvic acid. The cells were seeded 20 h before infection in 24-well culture plates at 3 × 10^5^ cells per well. Bacterial strains were grown overnight in LB (NaCl 5 g/l), diluted to 0.08 OD_580nm_ and grown for approximately 4 h more for *P. fluorescens* and 2 h more for *P. aeruginosa* to an OD_580nm_ between 1.0 and 1.5.

For the cytotoxicity assay, one day before infection, the macrophages were antibiotic starved. The macrophages were infected with bacteria resuspended in 1 ml of DMEM in order to give an MOI (multiplicity of infection) of 5 (15 × 10^5^ bacteria.mL^-1^). After 4 hours of incubation under controlled atmosphere (37°C, 5% CO_2_), lactate dehydrogenase (LDH) present in the supernatant was measured in each well using cytotox 96® enzymatic assay (Promega). LDH is a stable cytosolic enzyme released by eukaryotic cells and is an overall indicator of necrosis. J774A.1 cells exposed to Triton X100 (0.9%) were used as a control of total release (100% LDH release). The background level (0% LDH release) was determined with serum free culture medium. The percentage (%) of total lysis was calculated as follows: $\%=\left[\left(\text{X}-\text{B}\right)/\left(\text{T}-\text{B}\right)\right]\times 100$, where B (baseline) is a negative control and T (total lysis) is a positive control. X is the OD_490nm_ value of the analysed sample.

For *in vitro* microscopy, macrophages were infected with MFN1032 strain expressing Green Fluorescent Protein (pSMCP2.1 carrying *gfp* gene), resuspended in 1 ml of DMEM, in order to give an MOI of 10 and incubated for 10 min at 37°C, 5% CO_2_[37]. The medium was supplemented with 500 ng.mL^-1^ EtBr, which enters only into dead cells. Infection was followed using an inverted Zeiss (LSM 710) confocal laser-scanning microscope with an oil immersion 63X/1.40 plan-apochromatic objective. Plates were excited with a wavelength of 488 nm for GFP (emission: 493-539 nm) and 514 nm for EtBr (emission 589-797). 3D modelisation and orthographic representation were processed using Zen® 2009 (Zeiss) software and a Kernel of 3x3 (x, y) was applied.

### Expression of *rsc*STU genes from SBW25 in MFN1030 (MFN1032 hrpU-like operon disrupted mutant)

SBW25 was used for PCR amplification of *rsc*STU genes. PCR primers, *rsc*SSBW25 (5^′^-ATGGAACCAATCGATCTGTTC-3^′^) and SBW*rsc*U (5^′^-TCAGTGCCGTTCAAGCTC-3^′^), synthesized by Eurogentec (Angers, France), were designed to amplify *rsc*STU genes (2156 bp), a region of the *rsp* cluster I of SBW25, corresponding to genes *hrc*STU affected by hrpU-like operon disruption in MFN1030.

PCR was carried out in a 50 μL reaction volume, in a MJ mini thermal cycler (Bio-rad laboratories incorporation, USA). Reaction mixture contained 4 μL DNA, 0.5 μL Taq phusion polymerase (Biolabs, new England), 10 μL corresponding buffer, 4 μL primers (20 μM) and 4 μL deoxyribonucleoside triphosphate (2.5 mM). After initial denaturation for 10 seconds at 98°C, the reaction mixture was subjected to 30 cycles of 30 seconds at 98°C, 30 seconds at 49°C and 1 minute at 72°C, followed by a final 5 minutes extension at 75°C. Aliquots (10 μL) of the PCR products were analyzed by electrophoresis in 1% agarose gels, stained with ethidium bromide and photographed under UV illumination.

PCR product was cloned with the pBBR1MCS-5(4,8KB) digested by Sma I [38]. This construction, pBBR-*rsc*STU (6,9 kb), was then introduced into *Escherichia coli DH5α mcr* cells by electroporation. White colonies were selected for their resistance to gentamycin (20 μg/mL). Plasmids were isolated using the QIAprep Spin Miniprep Kit (Qiagen), checked by sequencing (beckman coulter genomics, Germany) and then transferred into the *Escherichia coli* conjugative strain S17.1.

MFN1030 (tetracyclin resistant) cells were conjugated with S17.1 cells carrying the pBBR-*rsc*STU plasmid and strains were selected for their resistance to tetracycline (20 μg.mL^-1^) and gentamycin (20 μg.mL^-1^). The resulting strain was called MFN1030-pBBR-*rsc*STU.

### Bacterial strains and culture conditions

The origin of each strain tested in this study can be found in Table 1. The bacteria were cultured in Luria Bertani medium (LB) at optimum growth temperatures, i.e. 28°C for *P. fluorescens* (for MF37 origin, see [39]) and *P. syringae* DC3000 [40], 37°C for *P. aeruginosa* CHA or PA14 [41,42] and *Klesiella aerogenes*[43], with shaking at 180 rpm. When necessary, 80 μg/mL Xgal, 20 μg/mL tetracycline, 20 μg/mL gentamycin or 30 μg/mL kanamycin were added. The bacterial density was determined by measuring optical density (OD) at 580 nm (Spectronic Unicam spectrophotometer).

## Authors’ contributions

DS carried out the assays with VD help and participated in the design of the manuscript. AM designed the study, wrote the manuscript and analyzed most of the data. LM and MH were involved in the *in vitro* microscopy assays and analysis. XL helped to design and writes the manuscript. NO and MF were involved in designing the study. All authors read and approved the final manuscript.
